# Human inflammatory dendritic cells in malignant pleural effusions induce Th1 cell differentiation

**DOI:** 10.1007/s00262-020-02510-1

**Published:** 2020-02-12

**Authors:** Fei-fei Gu, Jing-jing Wu, Yang-yang Liu, Yue Hu, Jin-yan Liang, Kai Zhang, Ming Li, Yan Wang, Yong-an Zhang, Li Liu

**Affiliations:** 1 Cancer Center, Union Hospital, Tongji Medical College, Huazhong University of Science and Technology, 1277 Jiefang Avenue, Wuhan, 430022 China; 2 Medical Oncology, Wuhan Pulmonary Hospital, 28 Baofeng Road, Wuhan, 430030 China; 3 Analysis and Testing Center, Institute of Hydrobiology, Chinese Academy of Sciences, 7 East Lake South Road, Wuhan, 430072 China; 4 College of Fisheries, Huazhong Agricultural University, 1 Shizishan Street, Wuhan, 430070 China

**Keywords:** Inflammatory DC, NSCLC, Pleural effusion, DC, T cell

## Abstract

Dendritic cells are crucial for the initiation and regulation of immune responses against cancer and pathogens. DCs are heterogeneous and highly specialized antigen-presenting cells. Human DCs comprise several subsets with different phenotypes and functional properties. In the steady state, human DC subsets have been well studied. However, the components of DC subsets and their immune functions during the inflamed setting are poorly understood. We identified and characterized DC subsets in the malignant pleural effusions of NSCLC patients. We analyzed the capacity of these DC subsets to induce T-cell differentiation. We observed the presence of inflammatory DCs (infDCs) and macrophages in the malignant pleural effusions of NSCLC patients, as identified by the CD11C^+^HLA-DR^+^CD16^−^BDCA1^+^ and CD11C^+^HLA-DR^+^CD16^+^BDCA1^−^ phenotypes, respectively. InfDCs represented approximately 1% of the total light-density cells in the pleural effusion and were characterized by the expression of CD206, CD14, CD11b, and CD1α, which were absent on blood DCs. InfDCs also expressed CD80, although at a low level. As infDCs did not express CD40, CD83 and CD275, they remained functionally immature. We found that TLR agonists promoted the maturation of infDCs. Compared with macrophages, infDCs had a weaker capacity to phagocytose necrotic tumor cell lysates. However, only infDCs induced autologous memory CD4^+^ T-cell differentiation into Th1 cells. For the first time, we found that infDCs were present in the malignant pleural effusions of NSCLC patients. We conclude that infDCs represent a distinct human DC subset and induce Th1 cell differentiation in the presence of TLR agonists.

**Electronic supplementary material:**

The online version of this article (10.1007/s00262-020-02510-1) contains supplementary material, which is available to authorized users.

## Introduction

Prior to the work of Ralph Steinman [1], the role of dendritic cells (DCs) was unknown. Since their initial discovery and naming by Ralph Steinman [1], the biological functions of DCs have been studied extensively [2–5]. As the predominant APC in humans, DCs play a key role in regulating immune responses. In addition, DCs play a crucial role in the regulation of central and peripheral immune tolerance [6].

In the steady state, DCs originate from hematopoietic progenitors in bone marrow and are a heterogeneous group of professional antigen-presenting cells present in small numbers. Human blood DCs comprise ~ 1% of the total PBMCs. According to their origins, DCs can be divided into two main lineages: plasmacytoid DCs (pDCs) and myeloid DCs (mDCs). The latter consists of CD141^+^ DC, CD16^+^ DC, and CD1c^+^ DC subsets [7–11]. However, a novel DC subset was found in mice in the inflamed setting, named inflammatory DCs (infDCs); these cells could not be found in steady-state tissue or lymphoid organs [12]. Initially, murine infDCs were defined as MHC-II^+^CD11b^+^F4/80^+^Ly6C^+^ DCs that differentiated from monocytes and migrated into inflammatory sites [12, 13]. Human infDCs, but not macrophages, can induce allogeneic CD4^+^ T-cell proliferation and express FcεRI uniquely [14]. However, the role of infDCs in tumor microenvironments remains to be determined.

In 2015, the incidence and mortality of lung cancer were ranked highest in China [15]. Lung cancer has become the most common malignant tumor and the leading cause of cancer-related death in China, with a poor 5-year survival rate of ~ 15% [16, 17]. NSCLC accounts for approximately 75–80% of all lung cancers and presents as a locally advanced or widely metastatic tumor in up to 40% of cases at the time of diagnosis [18]. Unfortunately, very limited advances have been made in antitumor therapy, including surgery, radiation, chemotherapy and targeted therapies for lung cancer. As many patients lack effective therapy options, it is necessary to explore the application of immunotherapy in lung cancer. Pleural effusion, which indicates a poor prognosis, is one of the most common late-stage physical signs in NSCLC [19]. However, whether APCs are present in the inflammatory pleural effusions and their immune functions are unclear. In the present study, we studied the phenotype and immune function of APCs in the pleural effusions of lung cancer patients. For the first time, we found that infDCs were also present in the malignant pleural effusions of NSCLC patients. InfDCs induce CD4^+^ Th1 cell differentiation after they are stimulated by TLR agonists.

## Patients and methods

### Cell isolation and culture

Samples of lung tumor pleural effusions from untreated patients were obtained from the Cancer Center, Union Hospital, Tongji Medical College, Huazhong University of Science and Technology. Cells were isolated after centrifugation on a Ficoll gradient (Haoyang, Tianjin, China), followed by cell sorting on a FACSAria III instrument (BD Biosciences, USA). Buffy coats were obtained from healthy volunteers. PBMCs were prepared by centrifugation on a Ficoll gradient. Cell purity was assessed by double staining of CD16^−^/CD1c^+^ for infDCs (> 96%) and CD16^+^/CD1c^−^ for macrophages (> 98%) (Fig. S1). DCs were cultured in X-VIVO15 medium (LONZA, Switzerland) supplemented with 5% human AB serum (Haoyang, Tianjin, China), 1% penicillin–streptomycin solution (Gibco, USA), 1% HEPES solution (Beyotime, China), 0.5% 2-ME (Merck, Germany) and 1% l-glutamine solution (Sigma-Aldrich, USA).

### Effect of TLR agonists on the maturation of infDCs

InfDCs or macrophages (2.0 × 10^4^) were stimulated with the following TLR ligands as previously described [20]: 3 μg/mL R848, 1 μg/mL LPS and/or 1 μg/mL CD40L (all Sigma-Aldrich, USA) or LPS. After 24 h, the expression of CD40, CD80 and CD86 on infDCs was evaluated by flow cytometry on a BD FACSAria III instrument.

### Flow cytometry antibodies

The following antibodies were used for flow cytometry: FITC anti-human CD3, CD16, and CD8a; PE anti-human CD4, CD8a, CD14, CD56, CD11b, FcεRI, CD274, CD275, and CCR7; APC-eFluor780 anti-human HLA-DR; PE-Cy7 anti-human CD11c, CD45RO, CD127, CD62L, and IL-4; PerCP-eFluor710 anti-human CD1c; APC anti-human IL-17A, CD45RA, CD206, TIGIT, CD172α, and CD209; PerCP-cy5.5 anti-human CD25 and CD44; PE-eFluor610 anti-human IFN-γ and human Fc blocking regiment(eBioscience, USA); FITC anti-human HLA-DR; PE anti-human CD1α, CD80, CD83, and CD86; APC anti-human 40; PE-Cy7 anti-human CD69 (BD Bioscience, USA); and APC anti-human CD103 (BioLegend, USA).

### Cell lines

The human NSCLC cell line HCC827 cells were cultured with complete RPMI-1640 medium containing 10% FBS (ScienCell, USA) and 1% penicillin–streptomycin (Gibco, USA).

### FITC-dextran uptake analysis

InfDCs or macrophages were inoculated into U-bottom 96-well plates (2.0 × 10^4^ cells/well/200 μL medium) and FITC-dextran (Sigma-Aldrich, USA) was added to each well of the experimental group to a final concentration of 100 μg/mL. For the control group, an equal volume of 1 × PBS buffer was added per well, and cells were cultured at 4 °C or 37 °C. After 1 h, the cells were harvested by digestion with 0.25% trypsin-0.53 mM EDTA solution (Gibco, USA). The percentage and MFI of FITC^+^ DCs were measured by flow cytometry on a BD FACSAria III instrument.

### Phagocytosis analysis of necrotic HCC827 cell lysates

The procedure utilized for phagocytosis has been described in detail previously [21, 22]. Briefly, for uptake experiments, HCC827 cells in the logarithmic growth phase were labeled with 10 μM PKH26 red fluorescent dye (Sigma-Aldrich, USA), according to the manufacturer’s instructions, and more than 99% of cells were stained (Fig. S2). Staining was followed by three cycles of rapid freezing at − 80 °C and thawing to induce cell necrosis. Then, 2.0 × 10^4^ PKH26^+^ HCC827 cells were cocultured with infDCs or macrophages at a 1:1 ratio in U-bottom 96-well plates at 4 °C for 12 h or 37 °C for 0.75, 1.5, 2, and 12 h and stained with mouse anti-human HLA-DR-FITC for analysis by flow cytometry or stained and analyzed by confocal microscopy.

For confocal microscopy, cells were labeled with HLA-DR-FITC before being fixed with 4% paraformaldehyde, allowed to dry on microscope slides and then mounted with DAPI (BioLegend, USA). Cells were viewed on a laser-scanning microscope (Leica SP8; Leica, Germany.) at 21 °C. Cell images were acquired using Leica SP8 software (version 2.6.3).

### T helper cell polarization

Pleural effusion memory CD4^+^ T cells and allogeneic CD4^+^ T cells were isolated by cell sorting on a FACSAria III instrument. Pleural effusion memory CD4^+^ T cells were gated as CD3^+^CD4^+^CD25^−^CD45RO^+^. Allogeneic CD4^+^ T cells from PBMCs from healthy donors were gated as CD3^+^CD4^+^. The PBMCs from healthy donors were obtained from our lab members. InfDCs or macrophages (2 × 10^4^ cells/well) were cultured with pleural effusion memory CD4^+^ T cells or allogeneic CD4^+^ T cells (1 × 10^5^ cells/well) in X-VIVO15 medium. For intracellular staining of pleural effusion memory CD4^+^ T cells or allogeneic CD4^+^ T cells, cells were analyzed after 18 h of culture in the presence or absence of LPS and R848 and after an additional 3 h in the presence of brefeldin A (Sigma). Cells were stained with FITC anti-CD3, fixed with IC Fixation Buffer and permeabilized with Permeabilization Buffer (eBioscience, USA) according to the manufacturer’s instructions. Cells were stained with PE-eFluor610 anti-human IFN-γ, PE-Cy7 anti-human IL-4 and APC anti-human IL-17A.

### Statistical analysis

Data obtained from BD FACSAria III and BD LSR II flow cytometers were analyzed using FlowJo V10 (Tree Star, USA). Paired data were subjected to a two-tailed Wilcoxon signed rank test using GraphPad Prism 6 statistical software. Unpaired data were analyzed by a two-tailed Mann–Whitney *U* test to determine the difference between the two groups. *P* < 0.05 was considered statistically significant. The bars and error bars in the bar graphs correspond to the mean and standard deviation, respectively, and the data shown are representative results obtained from 3 or more independent replicate experiments.

## Results

### Phenotype of the main immune cells in human malignant pleural effusions of NSCLC patients

**Fig. 1:**
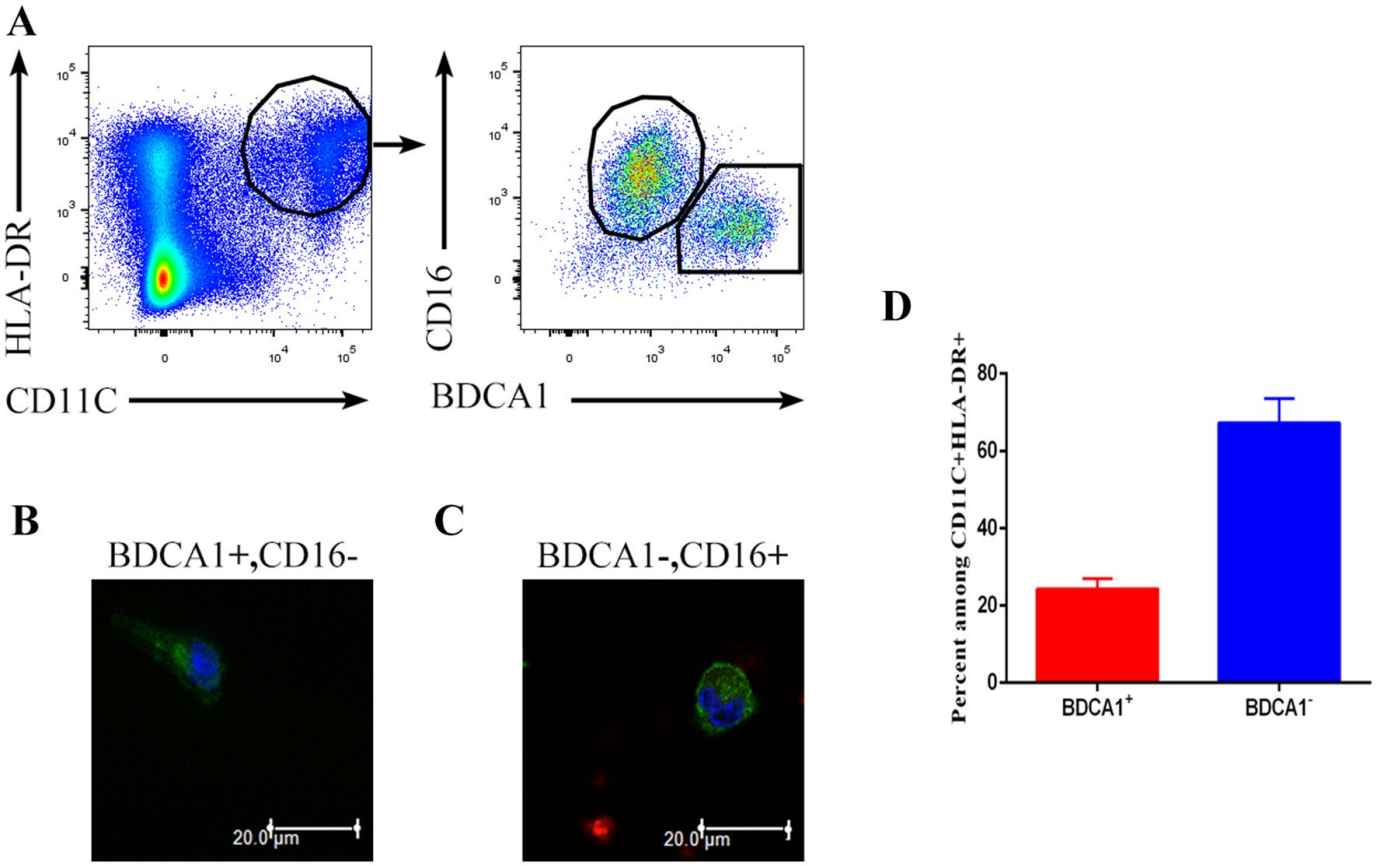
Identification of DCs in human malignant pleural effusions. **a** Light density cells from the malignant pleural effusions of NSCLC patients were stained with anti-HLA-DR, CD11C, CD16 and CD1c antibodies and analyzed by flow cytometry. One representative experiment out of 8 is shown. Sorted HLA-DR^+^ CD11c^+^ CD16^−^ BDCA1^+^ (**b**) and HLA-DR^+^ CD11c^+^ CD16^+^ BDCA1^−^ (**c**) cells from the malignant pleural effusions were analyzed by laser-scanning confocal microscopy. One representative experiment out of three is shown. **d** Percentage of CD16^−^BDCA1^+^ and CD16^+^BDCA1^−^ cells among the CD11C^+^HLA-DR^+^ cells from the malignant pleural effusion of NSCLC patients. The mean ± SD is shown (*n* = 12)

To investigate the major types of immune cells in the malignant pleural effusions of NSCLC patients, we analyzed malignant pleural effusion from untreated patients. We observed the presence of a cluster of double-positive (CD11C^+^HLA-DR^+^) cells, which mainly contained two populations: CD16^+^BDCA1^−^ cells and CD16^−^BDCA1^+^ cells (Fig. 1a). CD16^−^BDCA1^+^ cells had a long dendritic structure and were distinct from the macrophage-like CD16^+^BDCA1^−^ cells (Fig. 1b, c). These results are consistent with a study [14]. Therefore, the CD16^−^BDCA1^+^ cells were DCs, namely, infDCs, whereas the CD16^+^BDCA1^−^ cells were macrophages. In addition, CD16^−^BDCA1^+^ cells accounted for approximately 25% of the CD11C^+^HLA-DR^+^ cells in malignant pleural effusions (Fig. 1d).

**Fig. 2:**
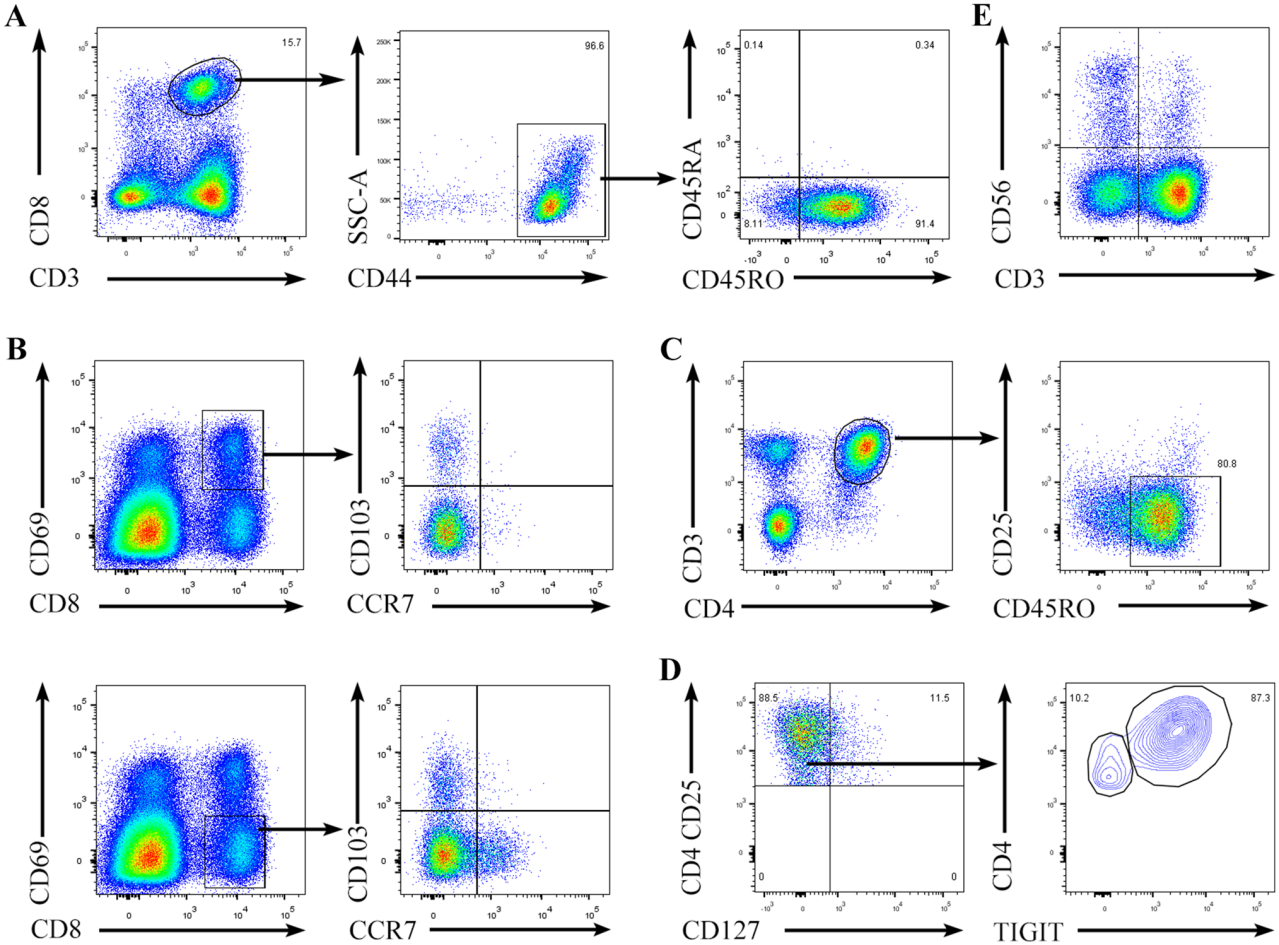
T cells and NK cells from the malignant pleural effusions. Light density cells from the malignant pleural effusions of NSCLC patients were stained with anti-CD8, CD3, and CD44 antibodies (**a**), anti-CD8, CD69, CD103, and CCR7 antibodies (**b**), anti-CD3, CD4, CD25, and CD45RO antibodies (**c**), anti-CD4, CD25, CD127, and TIGIT antibodies (**d**), or anti-CD3 and CD56 antibodies (**e**) and analyzed by flow cytometry. One representative experiment out of 4 is shown

Then, we performed phenotypic and component analyses of T cells and NK cells in the malignant pleural effusions. Our results revealed that more than 90% of the CD8^+^ T cells were CD45RA^−^CD45RO^+^ (Fig. S3), and almost all expressed the adhesion molecule CD44 (Fig. 2a). Based on the expression of CD44, CD69, CD103 and CCR7, CD8^+^ T cells could be divided into five main subsets: central memory T cells (TCMs) (CD44^high^CD69^−^CD103^−^CCR7^+^), effector memory T cells (TEMs) (CD44^high^CD69^−^CD103^−^CCR7^−^), tissue-resident memory T cells (TRMs) (CD44^high^ CD69^+^CD103^+^CCR7^−^) and other T cells (possibly effector T cells) (CD44^high^CD69^+^CD103^−^CCR7^−^ and CD44^high^CD69^−^CD103^+^CCR7^−^). Among these cells, memory CD8^+^ T cells represented approximately 70% of the total CD8^+^ T cells (Fig. 2a, b). In addition, CD4^+^ T cells were predominantly memory T cells, as shown by the expression of CD45RO and the absence of CD25 (Fig. 2c). A small number of CD4^+^ T cells were CD3^+^CD4^+^CD25^high^CD127^−^ Tregs, and the majority of Tregs expressed T-cell immunoreceptors with Ig and ITIM domains (TIGIT) (Fig. 2d). In addition, a small number of CD3^+^CD56^+^ natural killer T (NKT) cells and CD3^−^CD56^+^ NK cells were present in the malignant pleural effusions, and NK cells accounted for approximately 3% of the total mononuclear cells present (Fig. 2e).

### Characteristics of infDCs and macrophages in malignant pleural effusions

**Fig. 3:**
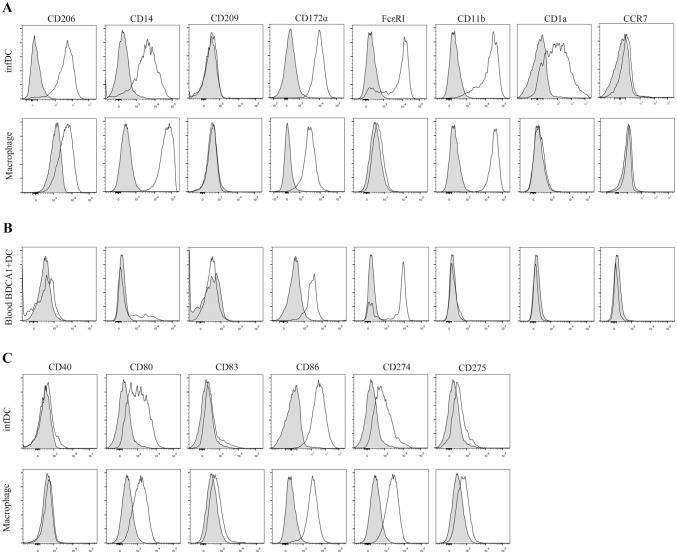
DCs from the malignant pleural effusions display a distinctive phenotype. **a** Light density cells from the malignant pleural effusions of NSCLC patients were stained with anti-HLA-DR, CD11C, CD16, CD1c and CD206, CD14, CD209, CD172α, FcεRI, CD11b, CD1a, CCR7 antibodies, or not stained as a control and analyzed by flow cytometry. DCs were gated as HLA-DR^+^ CD11C^+^ CD16^−^ CD1c^+^, and macrophages were gated as HLA-DR^+^ CD11C^+^ CD16^+^ CD1c^−^. One representative experiment out of 5 is shown. **b** Blood PBMCs were stained with anti-HLA-DR, CD11C, CD16, CD1c and CD206, CD14, CD209, CD172α, FcεRI, CD11b, CD1a, CCR7 antibodies, or not stained as a control and analyzed by flow cytometry. Blood DCs were gated as HLA-DR^+^ CD11C^+^ CD16^−^ CD1c^+^. The results are representative of three independent experiments. The gray histogram represents the blank control. **c** Light density cells from the malignant pleural effusions of NSCLC patients were stained with anti-HLA-DR, CD11c, CD16, CD1c and CD40, CD80, CD83, CD86, CD274, CD275, or not stained as a control and analyzed by flow cytometry. DCs were gated as HLA-DR^+^ CD11C^+^ CD16^−^ CD1c^+^, and macrophages were gated as HLA-DR^+^ CD11C^+^ CD16^+^ CD1c^−^. The results are representative of three independent experiments. The gray histogram represents the blank control

To gain insight into the specific phenotypes of infDCs and macrophages in malignant pleural effusion, we first compared their phenotypes with blood BDCA1^+^ DCs. Our results demonstrated that infDCs expressed CD11b, CD14, CD206, and CD172α (Sirpα), similar to macrophages in the malignant pleural effusions. In contrast, neither cell type expressed CD209 (DC-SIGN) or CCR7. InfDCs, but not macrophages, expressed FcεRI and CD1α (Fig. 3a). This phenotype was distinct from that of blood BDCA1^+^ DCs (Fig. 3b). Therefore, these results identify infDCs as being present in the malignant pleural effusions of NSCLC patients.

The direction of immunity depends on the condition of DCs; only mature DCs initiate immune responses, while immature DCs induce immune tolerance. Therefore, we further explored the maturity state of infDCs compared with that of macrophages. We found that infDCs and macrophages both expressed high levels of CD86 and low levels of CD80 and CD274 (PD-L1), but lacked CD40, CD83 and CD275 (ICOS-L) (Fig. 3c). Taken together, these results showed that both infDCs and macrophages were immature.

### TLR agonists promoted the maturation of infDCs

**Fig. 4:**
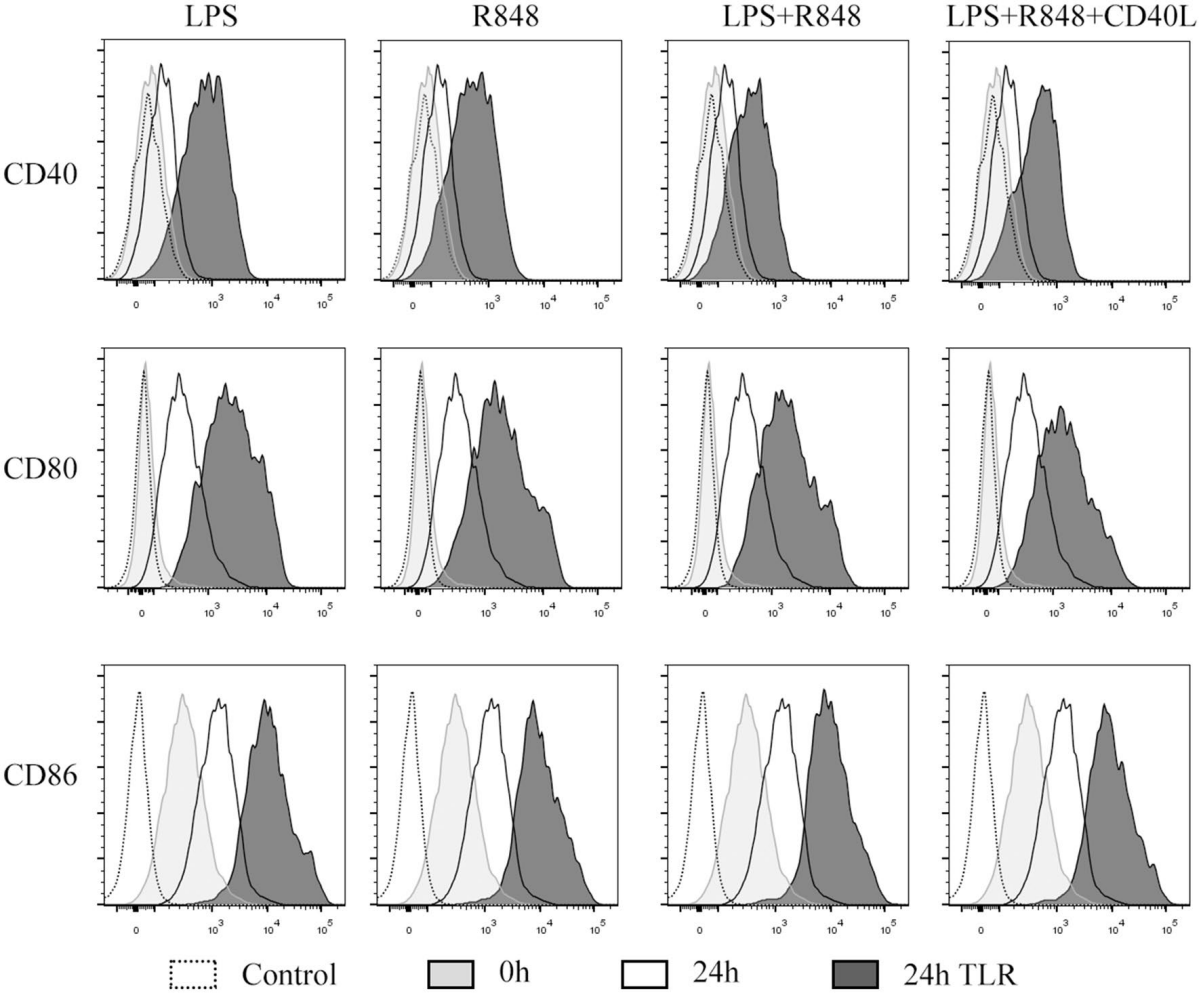
TLR agonists promote the expression of costimulatory molecules by infDCs. The expression of CD40, CD80, CD83, and CD86 was measured by flow cytometry after infDCs were cultured for 24 h in the presence or absence of 1 μg/mL LPS, 3 μg/mL R848, and/or 1 μg/mL CD40L. Representative results of three independent experiments

InfDCs secrete high levels of IL-12p70 after activation with a cocktail of CD40L and IFN-γ in the presence or absence of Pam3, which is a TLR1/2 ligand [14]. We speculated that infDCs would be matured by these cocktails and secrete more cytokines. Then, we analyzed the effects of TLR agonists on infDC maturation. Freshly isolated infDCs expressed the costimulatory molecule CD86 but lacked CD40 and CD80 expression. Incubation in culture medium alone or in the presence of TLR ligands and/or CD40L for 1 day upregulated costimulatory molecule expression on infDCs. Interestingly, we observed that the expression of CD86 was higher on infDCs cultured in medium alone than on infDCs cultured in the presence of TLR agonists and/or CD40L (Fig. 4). These data support the hypothesis that infDCs were immature and could mature via TLR ligands and/or CD40L.

### Exploration of the phagocytic function of infDCs

**Fig. 5:**
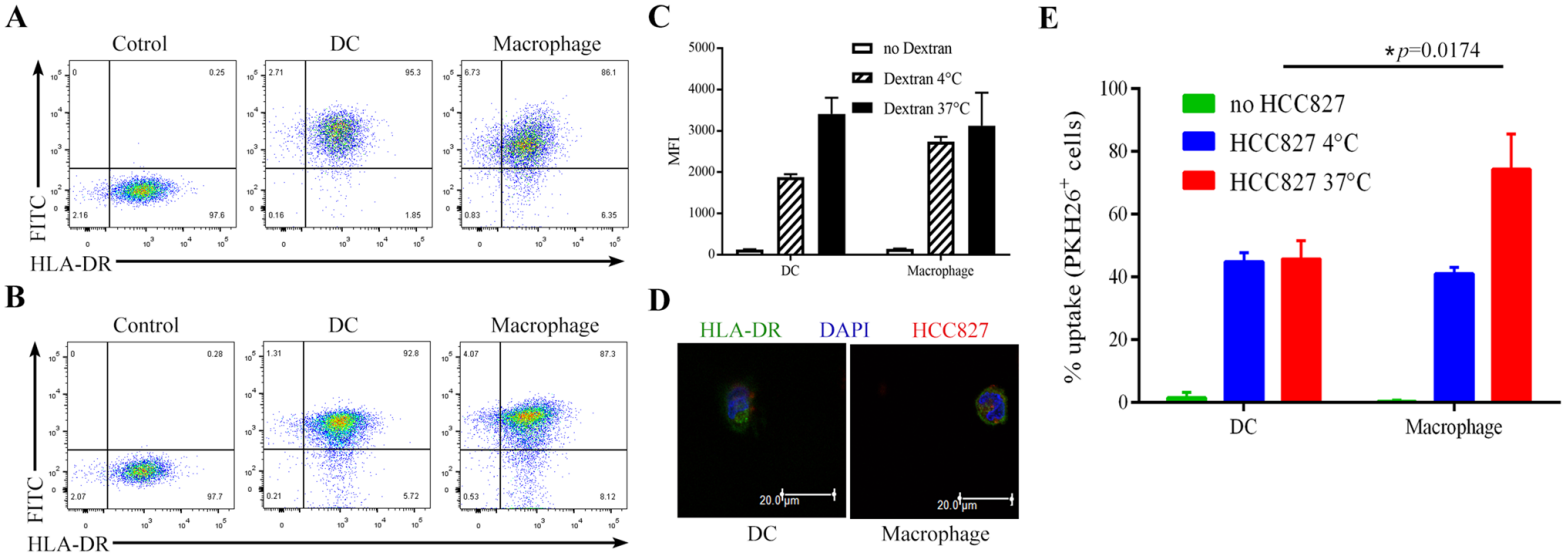
Uptake of FITC-dextran and necrotic lung cancer cells by infDCs and macrophages. Purified infDCs or macrophages (2 × 10^4^) were cultured for 1 h in the presence of 100 μg/mL FITC-dextran at 37 °C (**a**) or 4 °C (**b**), and the percentage of FITC^+^ infDCs and FITC^+^ macrophages was analyzed by flow cytometry. **c** The MFI of FITC^+^ infDCs and FITC^+^ macrophages was measured by flow cytometry. The mean ± SD is shown (*n* = 4). **d**. Purified infDCs or macrophages (2 × 10^4^) were incubated for 12 h with necrotic PKH26^+^HCC827 cells at a 1:1 ratio at 37 °C and analyzed by confocal microscopy. **e**. Purified infDCs or macrophages (2 × 10^4^) were incubated for 12 h with necrotic PKH26^+^HCC827 cells at a 1:1 ratio at 37 °C or 4 °C and analyzed by flow cytometry. The results from three independent experiments are shown

The results above revealed that the infDCs in the malignant pleural effusion were immature DCs. To examine mannose receptor-mediated endocytosis in infDCs, we used FITC-labeled dextran (MW 40 kDa) as a foreign macromolecule antigen. Our results suggested that the proportion of FITC^+^ DCs was higher than that of FITC^+^ macrophages (Fig. 5a, b). However, the FITC^+^ DCs and FITC^+^ macrophages had similar MFI levels (Fig. 5c). Taken together, these results support the notion that infDCs have strong phagocytic activity.

Numerous malignant cells were found in the pleural effusions of NSCLC patients so, we explored whether infDCs could phagocytose tumor cell lysates from necrotic lung cancer cells. We observed that both infDCs and macrophages phagocytosed tumor cell lysates (Fig. 5d); however, macrophages were much more effective than infDCs (Fig. 5e). Interestingly, unlike macrophages, infDCs had a similar phagocytosis capacity at 37 °C and 4 °C (Fig. 5e).

### InfDCs induced memory CD4^+^ T cells to differentiate into Th1 cells

**Fig. 6:**
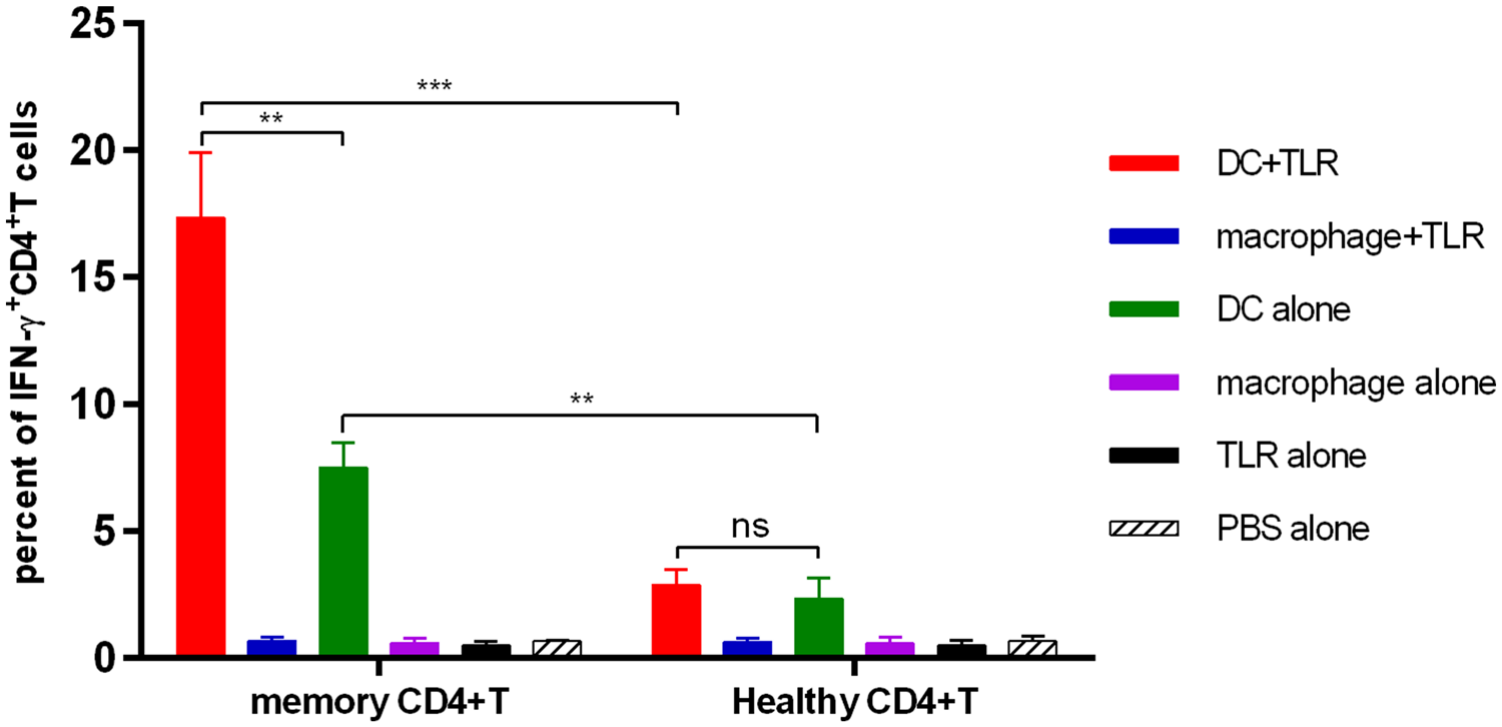
InfDCs induce Th1 cell differentiation. Purified infDCs or macrophages (2 × 10^4^/well) from the malignant pleural effusions were cultured for 21 h with autologous pleural effusion memory CD4^+^ T or allogeneic CD4^+^ T cells isolated from healthy donor PBMCs (1 × 10^5^/well) in the presence or absence of 1 μg/mL LPS and 3 μg/mL R848 and for an additional 4 h in the presence of brefeldin A, then fixed, permeabilized, and stained for IFN-γ. Results of three independent experiments are shown

In murine tissues, monocyte-derived DCs (MoDCs) can directly activate memory T cells [23]. InfDCs in tumor ascites induce autologous memory CD4^+^ T cells to differentiate into Th17 cells and Th1 cells [14]. Dendritic cells have strong plasticity; thus, different immune microenvironments may affect their immunologic function. Therefore, we analyzed the capacity of infDCs in pleural effusions from untreated NSCLC patients to induce autologous CD4^+^ T-cell differentiation. Unlike the results of past studies, our work showed that infDCs in malignant pleural effusions could only induce autologous memory CD4^+^ T cells to differentiate into Th1 cells, and the capacity was enhanced by LPS and R848 (Fig. 6). Neither autologous memory CD4^+^ T cells nor allogeneic CD4^+^ T cells produced detectable levels of IL-4 or IL-17A (unpublished data). These results show that compared to inflammatory macrophages, infDCs are potent stimulators of Th1 cells.

## Discussion

In this study, we first confirmed the presence of infDCs in the inflammatory pleural effusions of NSCLC patients. InfDCs had a distinctive phenotype, including several unique markers absent from conventional DCs. Compared with macrophages, infDCs are potent inducers of Th1 cells. These findings contribute to our understanding of infDCs in tumor microenvironments and provide a basis for future research.

Both human infDCs and mouse infDCs express CD11b, FcεRI, CD206 and CD172α [12, 14]. Mouse inflammatory DCs are differentiated from Ly6C^high^ monocytes recruited to the infection site [13, 24]. However, human infDCs may have a unique ontogeny, as human infDCs express some transcription factors involved in DC and macrophage development. In addition, infDCs and MoDCs share many common gene signatures [14]. Therefore, we speculate that monocytes rather than DC precursors are the direct precursors of human infDCs. The human CD14^+^ monocyte subset is the human functional equivalent of the mouse Ly6C^high^ monocyte subset [25]. However, whether human infDCs derive from CD14^+^ monocytes or CD16^+^ monocytes is unclear. Finally, Zbtb46 and CSF1R/CD115 play a crucial role in mouse infDC development [26, 27]. Interestingly, human infDCs also express high levels of Zbtb46 and CSF1R/CD115 [12]. Thus, we suggest that human infDCs and mouse infDCs most likely share a similar ontogeny.

Monocytes are generally considered to be very plastic cells. InfDCs originating from murine monocytes have been shown to induce Th1- [13, 28, 29] or Th2-mediated immune responses to different pathogens [30, 31], while human infDCs in tumor ascites and rheumatoid arthritis have a robust ability to preferentially induce Th17 cells [14]. However, this does not appear to be consistent with our findings. In our present study, infDCs in the malignant pleural effusions of NSCLC patients only induced Th1 cells, which might be explained by the fact that different inflammatory microenvironments form distinct infDCs with unique functions.

The Th1 cell-mediated immune response is the most important form of antitumor immunity. We have shown that infDCs have a strong capacity to take up soluble antigens and necrotic lung cancer cell lysates and induce Th1 cell differentiation in vitro. However, the immunosuppressive microenvironment, which is formed during the development of the tumor, inhibits the antitumor response induced by immune cells. Our data demonstrated that most of the Treg cells in the pleural effusions of lung cancer patients were characterized by high expression levels of TIGIT (an immunosuppressive molecule). Interestingly, TIGIT has been shown to enhance the immunosuppressive function of Treg cells [32]. Despite NK cells and NKT cells being present in malignant pleural effusions, a previous study confirmed that these NK cells could display strong antitumor activity until they were activated by short-term IL-2 administration [33]. In addition, infDCs were immature. In summary, the available data suggest that the malignant pleural effusions of lung cancer patients contain an immunosuppressive microenvironment; therefore, it is difficult to induce infDC antitumor immune responses in vivo. This study enhances our understanding of infDCs. However, what the capacity of infDCs is to cross-present tumor antigens and how to mature infDCs to promote their antitumor effect in vivo are important questions for future studies.

We first found a new DC subset in malignant pleural effusions of NSCLC patients. Although both DC subsets express BDCA1, infDCs and blood CD1c^+^ DCs are two different DC subsets. InfDCs induce Th1 cell differentiation after activation with TLR4, 7 and 8 agonists (LPS and R848). Our study provides important insights into DCs and extends our knowledge of immune cells in the malignant pleural effusions of lung cancer patients.

## Electronic supplementary material

Below is the link to the electronic supplementary material.

Supplementary file1 (PDF 200 kb)

## Data Availability

All data generated or analyzed during this study can be achieved from the correspondence author for reasonable reasons.

## Abbreviations

BDCA: Blood dendritic cell antigen
CCTCC: China Center for Type Culture Collection
FcεRI: Fc region of immunoglobulin E
infDC: Inflammatory dendritic cell
mDC: Myeloid DC
MoDC: Monocyte-derived DC
NKT: Natural killer T
pDC: Plasmacytoid DC
TCMs: Central memory T cells
TEMs: Effector memory T cells
TIGIT: T-cell immunoreceptor with Ig and ITIM domain
TRMs: Tissue-resident memory T cells

## References

[1] Steinman RM, Cohn ZA (1973). Identification of a novel cell type in peripheral lymphoid organs of mice. I. Morphology, quantitation, tissue distribution. J Exp Med.

[2] Steinman RM, Cohn ZA (1974). Identification of a novel cell type in peripheral lymphoid organs of mice. II. Functional properties in vitro. J Exp Med.

[3] Steinman RM, Lustig DS, Cohn ZA (1974). Identification of a novel cell type in peripheral lymphoid organs of mice. 3. Functional properties in vivo. J Exp Med.

[4] Steinman RM, Adams JC, Cohn ZA (1975). Identification of a novel cell type in peripheral lymphoid organs of mice. IV. Identification and distribution in mouse spleen. J Exp Med.

[5] Steinman RM, Kaplan G, Witmer MD, Cohn ZA (1979). Identification of a novel cell type in peripheral lymphoid organs of mice. V. Purification of spleen dendritic cells, new surface markers, and maintenance in vitro. J Exp Med.

[6] Liu YJ, Soumelis V, Watanabe N, Ito T, Wang YH (2007). TSLP: an epithelial cell cytokine that regulates T cell differentiation by conditioning dendritic cell maturation. Annu Rev Immunol.

[7] Merad M, Sathe P, Helft J, Miller J, Mortha A (2013). The dendritic cell lineage: ontogeny and function of dendritic cells and their subsets in the steady state and the inflamed setting. Annu Rev Immunol.

[8] Ueno H, Klechevsky E, Schmitt N, Ni L, Flamar AL (2011). Targeting human dendritic cell subsets for improved vaccines. Semin Immunol.

[9] Palucka K, Banchereau J (2012). Cancer immunotherapy via dendritic cells. Nat Rev Cancer.

[10] Anguille S, Smits EL, Bryant C, Van Acker HH, Goossens H (2015). Dendritic cells as pharmacological tools for cancer immunotherapy. Pharmacol Rev.

[11] Tel J, Schreibelt G, Sittig SP, Mathan TS, Buschow SI (2013). Human plasmacytoid dendritic cells efficiently cross-present exogenous Ags to CD8+ T cells despite lower Ag uptake than myeloid dendritic cell subsets. Blood.

[12] Segura E, Amigorena S (2013). Inflammatory dendritic cells in mice and humans. Trends Immunol.

[13] Leon B, Lopez-Bravo M, Ardavin C (2007). Monocyte-derived dendritic cells formed at the infection site control the induction of protective T helper 1 responses against Leishmania. Immunity.

[14] Segura E, Touzot M, Bohineust A, Cappuccio A, Chiocchia G (2013). Human inflammatory dendritic cells induce Th17 cell differentiation. Immunity.

[15] Chen W, Zheng R, Baade PD, Zhang S, Zeng H (2016). Cancer statistics in China, 2015. CA Cancer J Clin.

[16] Chen W, Zheng R, Zeng H, Zhang S (2015). Epidemiology of lung cancer in China. Thorac Cancer.

[17] Siegel RL, Miller KD, Jemal A (2018). Cancer statistics, 2018. CA Cancer J Clin.

[18] Thomas A, Hassan R (2012). Immunotherapies for non-small-cell lung cancer and mesothelioma. Lancet Oncol.

[19] Clive AO, Kahan BC, Hooper CE, Bhatnagar R, Morley AJ (2014). Predicting survival in malignant pleural effusion: development and validation of the LENT prognostic score. Thorax.

[20] Nizzoli G, Krietsch J, Weick A, Steinfelder S, Facciotti F (2013). Human CD1c+ dendritic cells secrete high levels of IL-12 and potently prime cytotoxic T-cell responses. Blood.

[21] Jongbloed SL, Kassianos AJ, McDonald KJ, Clark GJ, Ju X (2010). Human CD141+ (BDCA-3)+ dendritic cells (DCs) represent a unique myeloid DC subset that cross-presents necrotic cell antigens. J Exp Med.

[22] Chiang MC, Tullett KM, Lee YS, Idris A, Ding Y (2016). Differential uptake and cross-presentation of soluble and necrotic cell antigen by human DC subsets. Eur J Immunol.

[23] Wakim LM, Waithman J, van Rooijen N, Heath WR, Carbone FR (2008). Dendritic cell-induced memory T cell activation in nonlymphoid tissues. Science.

[24] Shortman K, Naik SH (2007). Steady-state and inflammatory dendritic-cell development. Nat Rev Immunol.

[25] Geissmann F, Jung S, Littman DR (2003). Blood monocytes consist of two principal subsets with distinct migratory properties. Immunity.

[26] Satpathy AT, Kc W, Albring JC, Edelson BT, Kretzer NM (2012). Zbtb46 expression distinguishes classical dendritic cells and their committed progenitors from other immune lineages. J Exp Med.

[27] Greter M, Helft J, Chow A, Hashimoto D, Mortha A (2012). GM-CSF controls nonlymphoid tissue dendritic cell homeostasis but is dispensable for the differentiation of inflammatory dendritic cells. Immunity.

[28] Nakano H, Lin KL, Yanagita M, Charbonneau C, Cook DN (2009). Blood-derived inflammatory dendritic cells in lymph nodes stimulate acute T helper type 1 immune responses. Nat Immunol.

[29] Iijima N, Mattei LM, Iwasaki A (2011). Recruited inflammatory monocytes stimulate antiviral Th1 immunity in infected tissue. Proc Natl Acad Sci USA.

[30] Hammad H, Plantinga M, Deswarte K, Pouliot P, Willart MA (2010). Inflammatory dendritic cells–not basophils–are necessary and sufficient for induction of Th2 immunity to inhaled house dust mite allergen. J Exp Med.

[31] Plantinga M, Guilliams M, Vanheerswynghels M, Deswarte K, Branco-Madeira F (2013). Conventional and monocyte-derived CD11b(+) dendritic cells initiate and maintain T helper 2 cell-mediated immunity to house dust mite allergen. Immunity.

[32] Blake SJ, Dougall WC, Miles JJ, Teng MW, Smyth MJ (2016). Molecular pathways: targeting CD96 and TIGIT for cancer immunotherapy. Clin Cancer Res.

[33] Vacca P, Martini S, Garelli V, Passalacqua G, Moretta L (2013). NK cells from malignant pleural effusions are not anergic but produce cytokines and display strong antitumor activity on short-term IL-2 activation. Eur J Immunol.

