# Supplementary material for: Human inflammatory dendritic cells in malignant pleural effusions induce Th1 cell differentiation
Source: Cancer Immunol Immunother. 2020 Feb 12;69(5):779–88. doi: 10.1007/s00262-020-02510-1 (PMC7183501; doi:10.1007/s00262-020-02510-1)
Supplement: Supplementary file 1 — Supplementary file1 (PDF 200 kb) [file 262_2020_2510_MOESM1_ESM.pdf]

**Supplementary data**

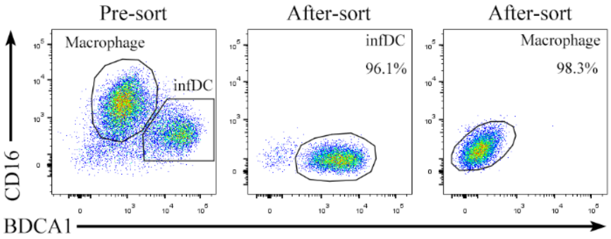


**Fig S1.** Purity of infDCs and macrophages sorted by flow cytometry. Light density cells from malignant pleural effusions were labeled with anti-HLA-DR, CD11c, CD16, CD1c antibodies and sorted by flow cytometry, and then the purity of these cells was detected.

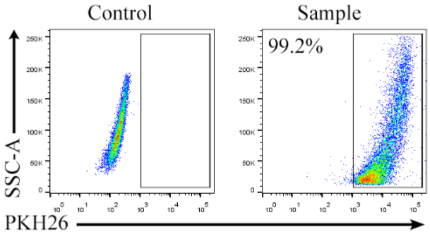


**Fig S2.** HCC827 cells stained with PKH26 red fluorescence. HCC827 cells were obtained, stained with PKH26 and analyzed by flow cytometry.

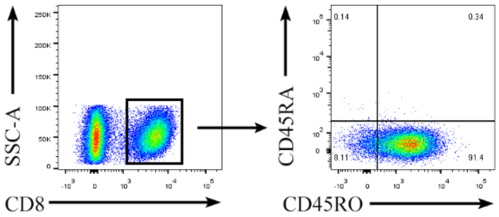


**Fig S3.** $CD8^+$ T cells from malignant pleural effusions. Light density cells from malignant pleural effusions of NSCLC patients were stained with anti-CD8, CD45RA, and CD45RO antibodies and analyzed by flow cytometry. One representative experiment out of 3 is shown.
